# Using the combined gene approach and multiple analytical methods to improve the phylogeny and classification of *Bombus* (Hymenoptera, Apidae) in China

**DOI:** 10.3897/zookeys.1007.34105

**Published:** 2020-12-30

**Authors:** Liu-Hao Wang, Shan Liu, Yu-Jie Tang, Yan-Ping Chen, Jie Wu, Ji-Lian Li

**Affiliations:** 1 Key Laboratory of Pollinating Insect Biology of the Ministry of Agriculture, Institute of Apicultural Research, Chinese Academy of Agricultural Science, Xiangshan, Beijing 100093, China Institute of Apicultural Research, Chinese Academy of Agricultural Science Beijing China; 2 College of Resources and Environmental Sciences, Henan Institute of Science and Technology, Xinxiang, Henan 453003, China Henan Institute of Science and Technology Xinxiang China; 3 United States Department of Agriculture (USDA) – Agricultural Research Service (ARS) Bee Research Laboratory, Beltsville, Maryland, USA Agricultural Research Service, United States Department of Agriculture Beltsville United States of America

**Keywords:** *
Bombus
*, China, monophyletic, phylogenetic relationships, six genes, subgenera

## Abstract

Bumble bees are vital to our agro-ecological system, with approximately 250 species reported around the world in the single genus *Bombus*. However, the health of bumble bees is threatened by multiple factors: habitat loss, climate change, pesticide use, and disease caused by pathogens and parasites. It is therefore vitally important to have a fully developed phylogeny for bumble bee species as part of our conservation efforts. The purpose of this study was to explore the phylogenetic relationships of the dominant bumble bees on the Tibetan plateau and in northern China as well as their placement and classification within the genus *Bombus*. The study used combined gene analysis consisting of sequence fragments from six genes, 16S rRNA, COI, EF-1α, Argk, Opsin and PEPCK, and the phylogenetic relationships of 209 *Bombus* species were explored. Twenty-six species, including 152 gene sequences, were collected from different regions throughout China, and 1037 gene sequences representing 183 species were obtained from GenBank or BOLD. The results suggest that the 209 analyzed species belong to fifteen subgenera and that most of the subgenera in *Bombus* are monophyletic, which is in accordance with conventional morphology-based classifications. The phylogenetic trees also show that nearly all subgenera easily fall into two distinct clades: short-faced and long-faced. The study is the first to investigate the phylogenetic placement of *Bombus turneri* (Richards), *Bombus opulentus* Smith, *Bombus pyrosoma* Morawitz, *Bombus longipennis* Friese, *Bombus minshanensis* Bischoff, and *Bombus lantschouensis* Vogt, all of which are widely distributed throughout different regions of China. The knowledge and understanding gained from the findings can provide a molecular basis to accurately classify *Bombus* in China and to define strategies to conserve biodiversity and promote pollinator populations.

## Introduction

Bumble bees belong to the genus *Bombus*, which has been classified in the tribe Bombini of the subfamily Apinae of the family Apidae. Four sister tribes including Bombini, Apini (e.g., honey bees), Meliponini (e.g., stingless bees), and Euglossini (e.g., orchard bees) belong to the corbiculate clade within the family Apidae (Winston and Michener 1977). With other species of bees and pollinators, bumble bees provide pollination services to vegetable crops in large greenhouses and to a great diversity of plants in the wild, and contribute substantially to the agriculture economy (Velthuis and Van Doorrn 2006; Carolan et al. 2012; Williams et al. 2012a) and biological diversity of ecosystems (Wang and Li 1998; Sun et al. 2003; He and Liu 2004; Goulson et al. 2008; An et al. 2011; Vergara and Fonseca-Buendía 2012). To date, there are approximately 250 known species of bumble bees in the world, with approximately 125 species documented in China (Cameron et al. 2007; Williams et al. 2010; An et al. 2011). However, with degradation of the ecological environment, human activity, pathogen infection and exposure to pesticides, populations of bumble bees are declining in China, especially in northwest China (Yang 1999; Xie et al. 2008), with some species even becoming extinct in certain areas (Rasmont et al. 2005; Williams 2005; Colla and Packer 2008; Goulson et al. 2008). Therefore, it is very important to know the distribution and phylogenetic evolution of bumble bee species in these regions, to be able to design effective conservation strategies for their protection.

The taxonomic status of closely related bumble bee taxa is often unclear. In the early twentieth century, we relied on morphological characters to classify *Bombus*. However, because of highly variable color patterns and the presence of convergent evolution in morphology, it is difficult to accurately identify the species within *Bombus* based only on morphological features (Hines and Williams 2012). Subsequently, male genitalia have been used to distinguish the different subgenera; they are more reliable than color pattern in classifying subgenera, although they do not unambiguously distinguish between species under certain circumstances (Stephen 1957; Thorp et al. 1983). With the development of molecular techniques, identification based on molecular markers has become a powerful tool in the phylogenetic analysis and placement of species of *Bombus* (Michener 2007). However, it is critical to choose appropriate genetic markers in molecular phylogenetic reconstructions. Over the years, the genes Cytochrome Oxidase subunit I (COI) and Cytochrome b (Cytb) have been used in phylogenetic studies on insects (Boehme et al. 2010; Wang and Qiao 2010). A specific COI fragment that is 648 bp long with enough genetic information and base variation has been used to effectively distinguish species (Hebert et al. 2003, 2004). COI has been widely applied to species identification and phylogenetics in insects (Pedersen 1996, 2002). Further, the mitochondrial gene 16S rRNA and nuclear genes elongation factor-1 alpha F2 copy (EF-1α), long-wavelength rhodopsin copy 1 (Opsin), arginine kinase (Argk), and phosphoenolpyruvate carboxykinase (PEPCK) have also been used for phylogenetic analysis (Kawakita et al. 2003, 2004; Cameron et al. 2007). The mitochondrial gene 16S rRNA is a useful marker for examining the phylogenetic position of some insects (Yoshizawa and Johnson 2003), and is the most informative for phylogenetic analysis of closely related species (Whitfield and Cameron 1998). EF-1α, which can promote aminoacyl-tRNA to combine with ribosomes, is often recognized as a good molecular marker to resolve the classification of insects at the phylogenetic level of family or genus (Cho et al. 1995; Mardulyn and Cameron 1999; Rokas et al. 2002; Danforth et al. 2004). The Opsin gene belongs to the family of the light absorption receptor proteins. It can distinguish evolutionary divergence in hymenopteran insects, including Cynipidae and Halictidae (Rokas et al. 2002; Danforth et al. 2004), and can be used to deduce the relationships between these and the corbiculate Apinae (Mardulyn and Cameron 1999; Cameron and Mardulyn 2001, 2003; Michel-Salzat and Whitfield 2004). ArgK is a kind of phosphate kinase which distributes broadly in the tissue of insects and is also a relatively conserved nuclear gene. The PEPCK sequence contains two parts, the high variation intron and the conserved exon, which are largely applied in the classification of the order Lepidoptera (Friedlander et al. 1996).

While advances in molecular marker techniques have led to significant improvements in population genetic analysis, the standard mitochondrial barcode fragment or nuclear genes are sometimes not informative enough to help understand the genetic variability of species. When multiple genes are combined for phylogenetic analysis, a much clearer view of the phylogeny among closely related species can be generated. Kawakita et al. (2004) elucidated the phylogeny of 65 species of bumble bees through the use of three nuclear genes, and analyzed their geographic distribution and character evolution. Later, Cameron et al. (2007) made a robust phylogeny with a comprehensive analysis of 219 species of bumble bees from all over the world, including some species from China, which utilized mitochondrial gene 16S rRNA and four nuclear gene sequences (Opsin, EF-1α, Argk, and PEPCK). Their results suggested that, overall, *Bombus* is monophyletic, with the subgenera grouped into two distinct clades, the short-faced and the long-faced, the first including a diverse New World clade. Their paper systematically analyzed the relationships among all subgenera and provided a foundation for the phylogeny of *Bombus*, although there remained some species that could not be included.

To improve our understanding of the phylogenetic relationships of *Bombus* in China, we conducted a phylogenetic analysis of 209 species by combining sequence fragments of two mitochondrial genes (COI and 16S rRNA) and four nuclear genes (EF-1α, Opsin, ArgK, and PEPCK). We obtained 152 gene sequences from 26 species recently collected from different regions of China. An additional 1093 gene sequences representing 183 additional species of *Bombus* were obtained from GenBank or BOLD. Among the 26 recently-collected species, *B. pyrosoma* Morawitz and *B. lantschouensis* Vogt are two common native species and important pollinators, characterized by having more workers in the colony, by ease of rearing in an indoor environment, and by a widespread distribution in China (Peng et al. 2009). Through this study, we have gained an insight into Chinese bumble bee species distributions and phylogenetic relationships, which in turn could be applied to our efforts of biodiversity conservation to promote pollinator populations.

## Materials and methods

### Bumble bee collection

**Figure 1.**
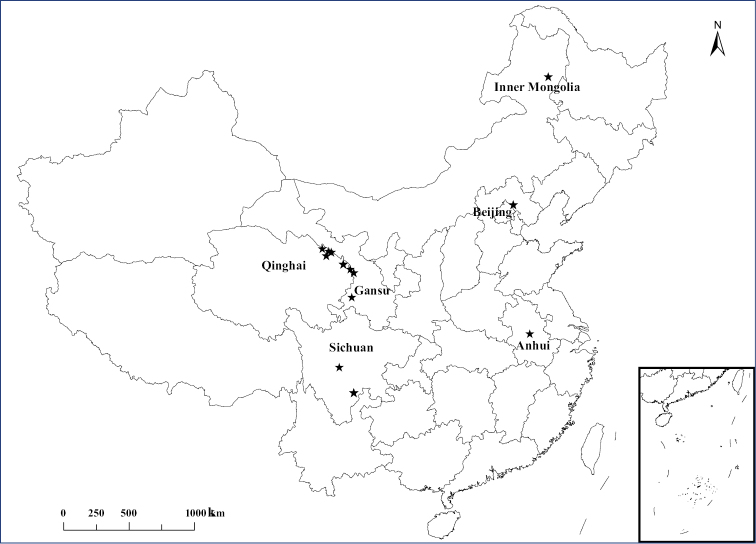
Sampling sites of *Bombus* spp. across different regions of China.

Bumble bee specimens were collected with nets and as a random sample at any given locality in the Sichuan, Inner Mongolia, Qinghai, Anhui, Gansu provinces and Beijing, China, between 2006 and 2012, and after capture were transferred directly into 100% ethanol. The samples were kept at -20 °C for subsequent analysis and voucher specimens are deposited at the Institute of Apicultural Research, Chinese Academy of Agricultural Science, Beijing, China. Exact collection localities are listed in Table 1 and shown in Fig. 1.

**Table 1.** *Bombus* samples collected during this study: classification, collectors, collection localities, voucher numbers, and GenBank accession numbers. “NA” indicates that the gene sequences could not be amplified by PCR.

Sample ID	Subgenus	Species	Province	Latitude and longitude	Altitude (m)	GenBank accession numbers					
						16S	COI	EF-1α	Opsin	Argk	PEPCK
BG104	*Alpigenobombus* Skorikov	*kashmirensis* Friese	Qinghai	37°59.967'N, 100°45.016'E	3214	KX791783	KX791757	KX791657	NA	KX791731	KX791707
BG017	*Bombus* s.s. Latreille	*longipennis* Friese	Qinghai	37°10.689'N, 102°03.091'E	2596	KX791770	KX791744	KX791644	KX791670	KX791720	KX791694
BG038		*patagiatus* Nylander	Inner Mongolia	48°41.426'N, 122°45.7'E	419	KX791787	KX791761	KX791661	KX791685	KX791735	KX791711
BG040		*lucorum* (Linnaeus)	Inner Mongolia	48°41.426'N, 122°45.7'E	419	KX791785	KX791759	KX791659	KX791683	KX791733	KX791709
BG054		*lantschouensis* Vogt	Gansu	36°34.728'N, 102°58.077'E	2096	KX791784	KX791758	KX791658	KX791682	KX791732	KX791708
BG177		*ignitus* Smith	Beijing	40°38.478'N, 117°15.188'E	314	KX791766	KX791740	KX791640	KX791666	KX791716	KX791690
BG191		*minshanensis* Bischoff	Gansu	34°54.161'N, 102°50.735'E	3138	KX791772	KX791746	KX791646	KX791672	KX791721	KX791696
BG133	*Megabombus* Dalla Torre	*trifasciatus* Smith	Sichuan	28°19.672'N, 103°07.999'E	2062	KX791778	KX791752	KX791652	KX791678	KX791727	KX791702
BG007	*Melanobombus* Dalla Torre	*supremus* Morawitz	Qinghai	37°41.413'N, 100°34.324'E	3146	KX791788	KX791762	KX791662	KX791686	KX791736	KX791712
BG029		*sichelii* Radoszkowski	Qinghai	37°10.689'N, 102°03.091'E	2596	KX791777	KX791751	KX791651	KX791677	KX791726	KX791701
BG078		*rufofasciatus* Smith	Qinghai	38°09.402'N, 100°11.705'E	2912	KX791776	KX791750	KX791650	KX791676	KX791725	KX791700
BG095		*ladakhensis* Richards	Qinghai	37°56.211'N, 100°57.941'E	3418	KX791768	KX791742	KX791642	KX791668	KX791718	KX791692
BG146		*friseanus* Skorikov	Sichuan	28°19.672'N, 103°07.999'E	2062	KX791765	KX791739	KX791639	KX791665	KX791715	KX791689
BG179		*pyrosoma* Morawitz	Beijing	40°38.478'N, 117°15.188'E	314	KX791774	KX791748	KX791648	KX791674	KX791723	KX791698
BG141		*festivus* Smith	Sichuan	28°19.672'N, 103°07.999'E	2062	KX791764	KX791738	KX791638	KX791664	KX791714	KX791688
BG003	*Mendacibombus* Skorikov	*waltoni* Cockerell	Qinghai	37°41.413'N, 100°34.324'E	3146	KX791789	KX791763	KX791663	KX791687	KX791737	KX791713
BG167	*Psithyrus* Lepeletier	*turneri* (Richards)	Anhui	31°49.021'N, 117°14.281'E	1700	KX791779	KX791753	KX791653	NA	KX791728	KX791703
BG025	*Pyrobombus* Dalla Torre	*lepidus* Skorikov	Qinghai	37°10.689'N, 102°03.091'E	2596	KX791769	KX791743	KX791643	KX791669	KX791719	KX791693
BG137		*flavescens* Smith	Sichuan	28°19.672'N, 103°07.999'E	2062	KX791782	KX791756	KX791656	KX791681	KX791730	KX791706
BG028	*Subterraneobombus* Vogt	*personatus* Smith	Qinghai	37°10.689'N, 102°03.091'E	2596	KX791773	KX791747	KX791647	KX791673	KX791722	KX791697
BG049		*melanurus* Lepeletier	Gansu	36°34.728'N, 102°58.077'E	2096	KX791771	KX791745	KX791645	KX791671	NA	KX791695
BG093		*difficillimus* Skorikov	Qinghai	37°56.211'N, 100°57.941'E	3418	KX791780	KX791754	KX791654	KX791679	NA	KX791704
BG060	*Thoracobombus* Dalla Torre	*filchnerae* Vogt	Gansu	36°49.855'N, 102°39.003'E	2210	KX791781	KX791755	KX791655	KX791680	KX791729	KX791705
BG153		*impetuosus* Smith s. l.	Sichuan	28°19.672'N, 103°07.999'E	2062	KX791767	KX791741	KX791641	KX791667	KX791717	KX791691
BG155		*remotus* (Tkalců)	Sichuan	30°02.905'N, 101°58.049'E	2833	KX791775	KX791749	KX791649	KX791675	KX791724	KX791699
BG172		*opulentus* Smith	Beijing	40°38.478'N, 117°15.188'E	314	KX791786	KX791760	KX791660	KX791684	KX791734	KX791710

### Morphology

All species were identified according to the morphological characters of bumble bees as described by Williams (1998). Subgenera and species were authenticated by the characters of the genitalia and other key taxonomic characters such as body size, color pattern, and leg structure (Williams et al. 2009; An et al. 2014). The detailed morphological classification is presented in Suppl. material 1.

### Genomic DNA extraction

For the extraction of nucleic acid, the muscle tissue of each individual bee’s thorax was cleanly cut off with scissors, immediately put into an aseptic tube and ground in liquid nitrogen with a pestle. DNA was extracted from bee muscle tissue using a Wizard^®^Genomic DNA Purification Kit (A1120, Promega). DNA extracts were kept at -20 °C until needed as a DNA template for the PCR.

### PCR amplification and sequencing

The specific primers used to amplify the two mitochondrial genes (COI and 16S rRNA) and four nuclear genes (Opsin, EF-1α, Argk, and PEPCK) are shown in Table 2. PCR reactions were performed using a Mastercycler 5333 (Eppendorf) in 25 μL PCR Mix (2×), 2 μL template genomic DNA (about 50 ng), 1 μL of each primer (forward and reverse), 21 μL ddH_2_O, with a final volume of 50 μL. PCR parameters for amplification were as follows: initial denaturation at 94 °C for 3 min, followed by 35 cycles denaturation at 94 °C for 1 min, annealing at 50–60 °C for 1 min, elongation at 72 °C for 1 min and final elongation at 72 °C for 10 min. The annealing temperatures for each gene were: 50 °C for PEPCK and Argk, 53 °C for EF-1α, 55 °C for 16S rRNA, 56 °C for COI, and 60 °C for Opsin. PCR products were electrophoresed in 1.2% agarose gel containing 0.5 µg/ml GoldView (GV) and visualized under UV light. PCR products were purified and then sent to Invitrogen for sequencing. After manual editing and error checking, we then performed a BLAST database search in GenBank to identify and include the closest matches of the same sequence for *Bombus* taxa. We obtained 152 valid sequences belonging to 26 *Bombus* species. The sequences used in this analysis have been deposited in GenBank. The list of sequences with their codes and the respective GenBank accession numbers can be found in Table 1.

**Table 2.** Primer information for the six genes used in this study.

Gene	Primer sequence (5'→3')	Reference
COI	ATTCAACCAATCATAAAGATATTGG (LepF)	Hebert et al. (2004)
	TAAACTTCTGGATGTCCAAAAAATCA (LepR)	
16S rRNA	CACCTGTTTATCAAAAACAT (16S Wb)	Williams et al. (2011, 2016)
	TATAGATAGAAACCAATCTG (16SIR)	
Opsin	AATTGCTATTAYGARACNTGGGT (Opsin-F)	Lin and Danforth (2004)
	ATATGGAGTCCANGCCATRAACCA (Opsin-R)	
EF-1α	GGRCAYAGAGATTTCATCAAGAAC (F2-ForH)	Franklin (1954)
	TTGCAAAGCTTCRKGATGCATTT (F2-RevH2)	
Argk	GTTGACCAAGCYGTYTTGGA (Argk1-F)	Kawakita et al. (2003)
	CATGGAAATAATACGRAGRTG (Argk1-R)	
	GACAGCAARTCTCTGCTGAAGAA (Argk2-F)	
	AGAACAATTATCTYAAATRCTAARCTTC (FHv5-F)	
	GGTYTTGGCATCGTTGTGGTAGATAC (Argk2-R)	
PEPCK	GTSTCTTATGGGAGSGGTTACGG (FH2-F)	Michel-Salzat and Whitfield (2004)
	TGTATRATAATTCGCAAYTTCAC (FHv4-F)	
	CTGCTGGRGTYCTAGATCC (RHv4-R)	

### Sequence analysis and construction of the phylogenetic tree

Altogether, 1245 gene sequences were used to conduct the phylogenetic analysis. One hundred and fifty-two (152) sequences from 26 bumble bee species collected during this study (Tab. 1) and 1037 sequences from 183 bumble bee species retrieved from GenBank or BOLD (see Cameron et al. 2007) were used to construct the phylogenetic tree. The Apini*Apis mellifera* Linnaeus and *Apis dorsata* Fabricius, the Meliponini*Liotrigona mahafalya* Brooks & Michener, *Heterotrigona itama* Cockerell, *Plebeia frontalis* Friese, *Trigona amazonensis* Ducke, *Geniotrigona thoracica* Smith and *Hypotrigona gribodoi* (Magretti) and the Euglossini*Eulaema boliviensis* (Friese) and *Euglossa imperialis* Cockerell were used as outgroups, as in Cameron et al. (2007).

The sequence data were aligned by ClustalX using default settings and visually checked using BioEdit (V7.0.9.0). We referred to Cameron et al. (2007), who had submitted the sequences to GenBank, and downloaded sequences of 16S rRNA, ArgK, EF-1α, Opsin, PEPCK and COI of bumble bees and outgroups from GenBank or BOLD. Phylogenetic analysis was conducted in MEGA 6.0 (Tamura et al. 2013).

Phylogenetic relationships were estimated by Bayesian analysis, maximum likelihood (ML) analysis, maximum parsimony (MP) analysis and Neighbor Joining (NJ) analysis, separately. Model selection for each gene was based on the Akaike Information Criterion (AIC) in Modeltest (Posada and Crandall 1998) and MrModeltest (Nylander 2004); the best model parameters for each gene partition were GTR+I+G. Bayesian analysis was performed using MrBayes v. 3.2.6 (Ronquist et al. 2012). Two independent Markov Chain Monte Carlo (MCMC) runs were conducted for 10 million generations, sampling every 1000 generations. Tracer v.1.6 was used to establish the convergence between two runs (Rambaut et al. 2014). Burn-in samples, which were the first 25% of the yielded trees, were discarded, then the remaining trees were used to generate a majority-rule consensus tree with posterior probabilities (PP). ML analysis were conducted using the GTRGAMMAI model of RAxML v.7.2.6. Node support was assessed via 1000 bootstrap replicates (Stamatakis 2006). MP analysis was performed using PAUP* v.4.0a165 (Swofford 2002). The tree bisection reconnection (TBR) branch swapping algorithm was used, and 100 random addition replicates were performed using heuristic strategy. Support values were assessed under the heuristic search with TBR and 100 jackknife replicates each with 100 random addition searches. NJ analysis was performed using MEGA v. 6.0 (Tamura et al. 2013). The phylogenetic trees were displayed and edited utilizing Figtree v.1.4.0.

## Results and discussion

### Phylogenetic analysis

**Figure 2.**
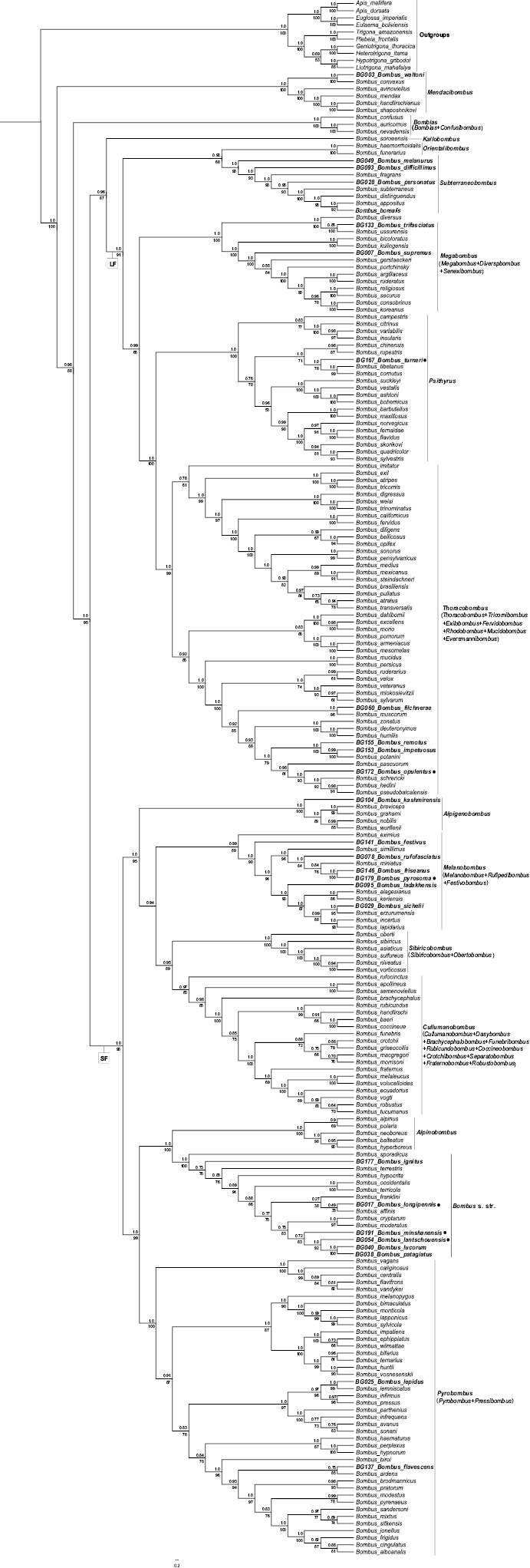
Estimated phylogeny of *Bombus* based on six combined gene sequences (mitochondrial genes 16S rRNA and COI, nuclear genes Opsin, ArgK, EF-1α, and PEPCK) analyzed by Bayesian Inference and Maximum Likelihood. Subgeneric clades are noted at the right of the figure, values above branches are posterior probabilities (BI), values below branches are bootstrap values (ML). Species in bold font were collected by the authors in China and a black spot indicates species that were not included in the previous phylogeny of *Bombus* of Cameron et al. (2007). The outgroups are at the top of the tree. Abbreviations: SF, short-faced clade; LF, long-faced clade. Subgenera that were synonymized are in parentheses.

**Figure 3.**
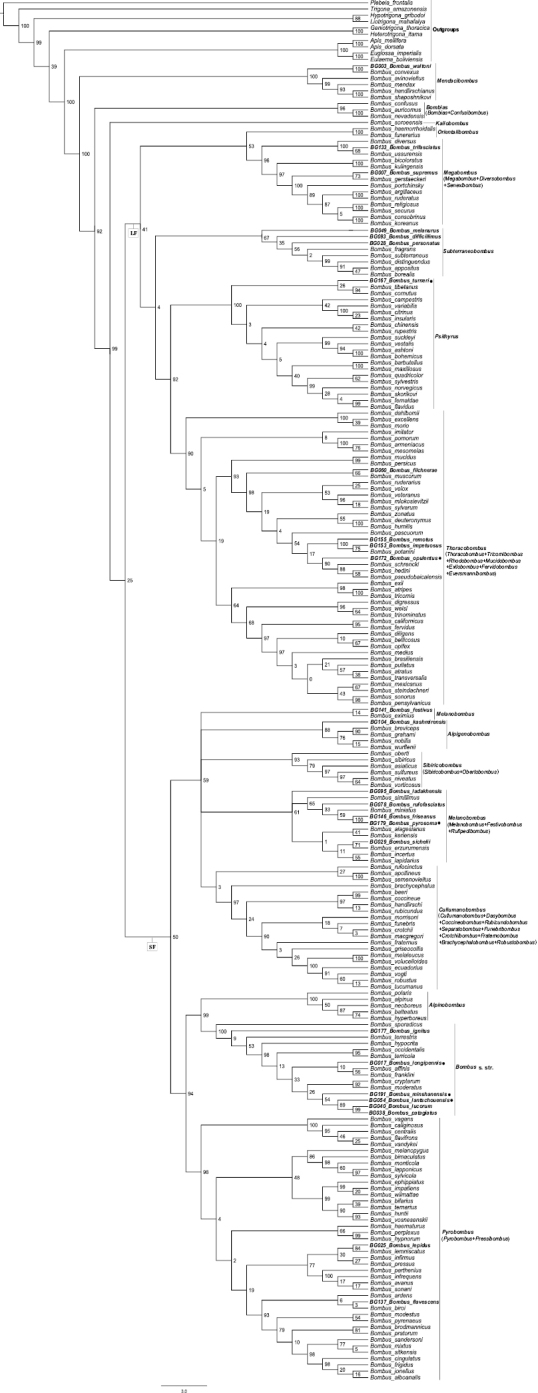
Estimated phylogeny of *Bombus* based on six combined gene sequences (mitochondrial genes 16S rRNA and COI, nuclear genes Opsin, ArgK, EF-1α, and PEPCK) analyzed by Maximum Parsimony. Subgeneric clades are noted at the right of the figure and values on branches are the bootstrap values. Species in bold font were collected in China and a black spot indicates species that were not included in the phylogeny of *Bombus* of Cameron et al. (2007). The outgroups are at the top of the tree. Abbreviations: SF, short-faced clade; LF, long-faced clade. Subgenera that were synonymized are in parentheses.

The results of the phylogenetic analysis of 209 *Bombus* species and ten outgroup species showed the same topology structure in two trees, which is similar to results in Cameron et al. (2007). The *Bombus* genus was divided into two distinct clades by Bayesian Inference (BI), ML and MP: the short-faced and the long-faced (Figs 2, 3). The morphological differences between the short-faced and long-faced clades are based on characters of the head including the length of the tongue, and on the presence/absence of a mid-basitarsal spine. The short-faced species are generally short-tongued without a mid-basitarsal spine, while the long-faced species are long-tongued with a mid-basitarsal spine. The subgenera *Mendacibombus*, *Confusibombus*, *Bombias*, and *Kallobombus* are separated into two clades, which is consistent with previous studies (Williams 1994; Pedersen 2002; Kawakita et al. 2003, 2004; Cameron et al. 2007) and well supported by posterior probability (PP) and bootstrap values. This is the first report on the phylogenetic evolution and classification status of *B. turneri* (Richards), *B. opulentus* Smith, *B. pyrosoma*, *B. longipennis* Friese, *B. minshanensis* Bischoff, and *B. lantschouensis*, which were collected from the Anhui, Qinghai, and Gansu provinces and Beijing in China, whereas *B. longipennis*, *B. minshanensis* and *B. lantschouensis* were revised by Williams et al. (2012b). Combining morphological data (Suppl. material 1) with the previous studies on *Bombus* taxonomy (Williams et al. 2009; An et al. 2014), we conclude that 1) *B. turneri* belongs to the subgenus Psithyrus, 2) *B. opulentus* is one of the species in the subgenus Thoracobombus, 3) *B. pyrosoma* belongs to *Melanobombus*, and 4) *B. longipennis*, *B. minshanensis*, and *B. lantschouensis* are grouped into *Bombus* s. str. These species are widely distributed throughout China. *Bombus longipennis* is distributed mainly in the medium elevation of mountains and on the Tibetan plateau in China, with a yellow-banded color pattern that is quite similar to that of *B. lucorum* (Linnaeus) and *B. cryptarum* (Fabricius). Therefore, *B. longipennis* has been confused with *B. lucorum* (An et al. 2014). *Bombus minshanensis* is primarily distributed in the medium to high elevations of the east Tibetan plateau meadows in China and has often been confused with *B. patagiatus* Nylander because of the similar white-banded color pattern in the females (An et al. 2014). *Bombus lantschouensis* is a common bumble bee species widely distributed in low, medium, and high elevation river valleys, mountains, and plateaus in China. *Bombus lantschouensis* has also been confused with *B. patagiatus*, as both species share the similar yellow-banded color pattern (An et al. 2014). *Bombus pyrosoma* is easily misidentified as *B. validus* Friese because of the similar white band (An et al. 2014). *Bombus turneri* is a parasitic bumble bee species and has a very small population, being found only in the medium elevations of the edge of the east Qinghai-Tibetan plateau and the loess plateau in China (An et al. 2014). *Bombus opulentus* has a dominant distribution in the low and medium elevations of mountains and plateaus in China. The brown and black color pattern of this species is similar to that of *B. longipes* Friese (An et al. 2014). These consistencies between molecular phylogeny and morphological classification have furthered knowledge of the distribution and evolutionary history of *Bombus* in China.

Our phylogeny is consistent with the studies reported by Cameron et al. (2007) in terms of the number and variety of subgenera in the short-faced and long-faced clades and the relationships among the different subgenera. We added six new species of *Bombus* from China into the trees. As a result, there were some differences in the subgenera *Psithyrus*, *Thoracobombus*, *Melanobombus* and *Bombus* s. str.; *B. lantschouensis* is sister to *B. lucorum* and *B. patagiatus*; *B. longipennis* and *B. affinis* Cresson were not well supported by BI and ML methods (PP = 0.49; bootstrap values = 33) in the *Bombus* s. str. clade, and *B. minshanensis* together with *B. lantschouensis* and *B. lucorum* + *B. patagiatus* formed a branch in the *Bombus* s. str. clade (Fig. 2).

Besides 20 species of bumble bees in our samples which were also included in the phylogenetic trees of Cameron et al. (2007), we replaced the original sequences in Cameron et al.’s study with new sequences generated from this study and reconstructed the phylogenetic trees (Figs 2, 3). The results were consistent with Cameron et al.’s phylogeny for most of the species, but there were some variations in the placements of *B. kashmirensis* Friese, *B. rufofasciatus* Smith, *B. friseanus* Skorikov, *B. lucorum*, and *B. patagiatus*. For example, *B. kashmirensis* was sister to *B. nobilis* Friese in Cameron et al.’s analysis, while it was placed in a single clade and grouped with four other species, *B. breviceps* Smith, *B. grahami* (Frison), *B. nobilis*, and *B. wurflenii* Radoszkowski, in our study. Also, *B. rufofasciatus* was sister to *B. miniatus* Bingham and grouped with *B. friseanus* + *B. formosellus* (Frison) in Cameron et al.’s study, but in our study it grouped with *B. miniatus* Bingham, *B. friseanus*, and *B. pyrosoma*. In *Bombus* s. str., *B. lucorum* is sister to *B. franklini* (Frison) and *B. patagiatus*, whereas it was sister to *B. cryptarum* (Fabricius) in Cameron et al.’s phylogeny. Furthermore, *B. lucorum* was sister to *B. patagiatus* in Cameron et al.’s study, but grouped with *B. lantschouensis* in our study. These differences may be due to the combined gene approach or to the addition of new species sequences in our study, and further research is needed to clarify this problem.

**Figure 4.**
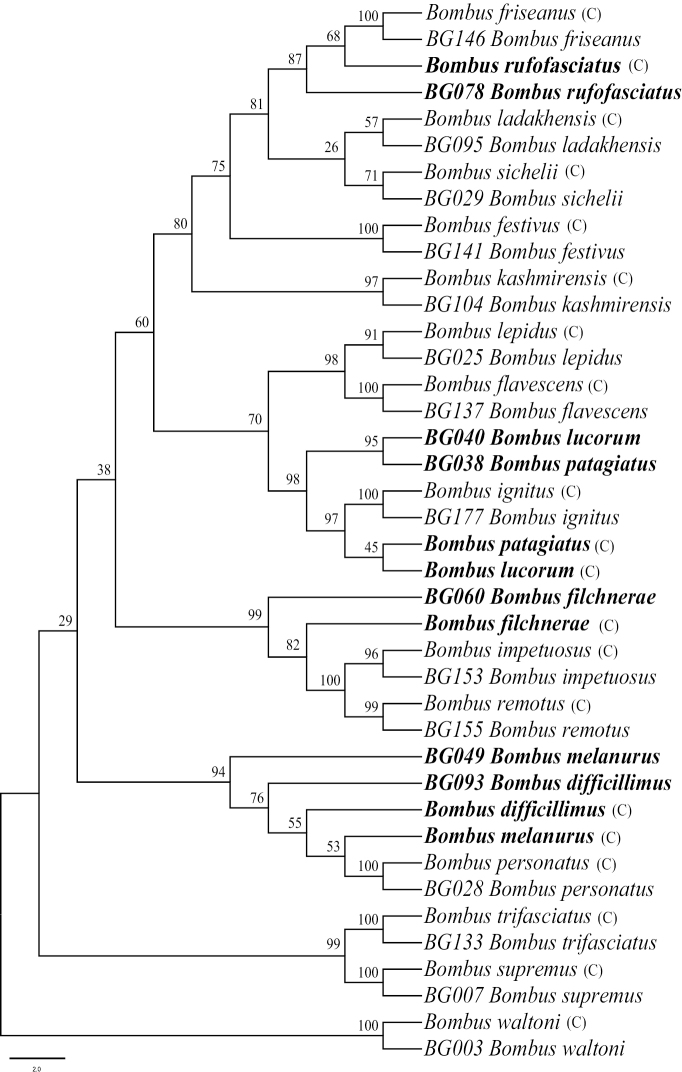
Estimated phylogeny of the same samples using both new sequences and sequences from Cameron et al. (2007), based on five combined gene sequences (mitochondrial gene 16S rRNA, nuclear genes Opsin, ArgK, EF-1α, and PEPCK) analyzed by Maximum Likelihood. Values on the branches are the bootstrap values, (C) represents Cameron et al.’s species, and “BG” represents our species. The bold font indicates species from both datasets that did not cluster together into monophyletic clades.

Based on the sequences of five genes (16S rRNA, Argk, EF-1α, Opsin, and PEPCK), we analyzed the relationships between the same 20 species and built one phylogenetic tree using the ML analysis (Fig. 4). In general, the results suggested that most species of *Bombus* were stable in genetic evolution and that their taxonomic positions showed no significant change with the variation of distribution areas. The results showed that nearly all specimens of the same species formed one clade in the phylogenetic tree (Fig. 4). We compared all gene sequences of the six species from Cameron et al. (2007) to our samples and found that there were some sequence variations between both groups of samples, which may reflect the adaptation to different geographical environments and evolutionary pathways in certain bumble bee species.

**Figure 5.**
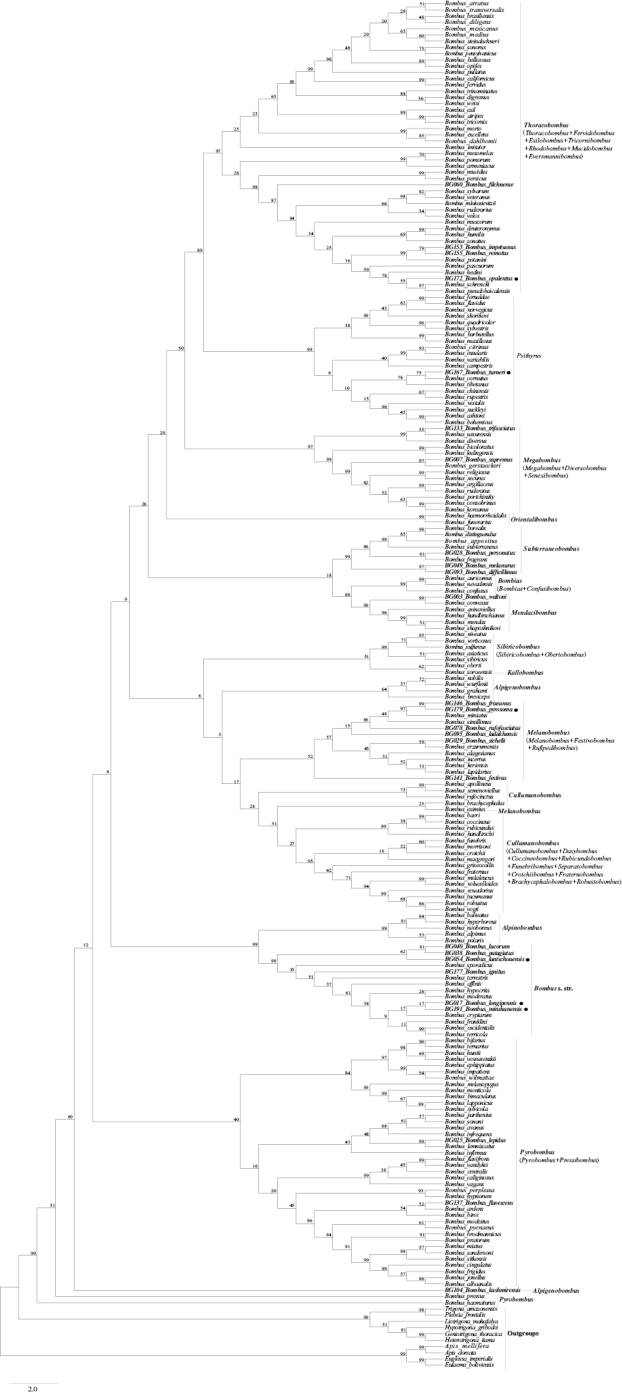
Estimated phylogeny of *Bombus* based on six combined gene sequences (mitochondrial genes 16S rRNA and COI, nuclear genes Opsin, ArgK, EF-1α, and PEPCK) analyzed by Neighbor Joining. Subgeneric clades are noted at the right of the Figure, and values on branches are the bootstrap values of NJ. Species in bold font were collected in China and a black spot indicates species that were not included in the phylogeny of *Bombus* of Cameron et al. (2007). The outgroups are at the bottom of the tree. Subgenera that were synonymized are in parentheses.

To ensure the accuracy in the classification of species using the combined gene approach, we utilized six genes to analyze the relationships among species. In Cameron et al. (2007), *B. ruderarius* (Müller) and *B. velox* (Skorikov) were sister species supported by PP = 0.52, but in our analysis *B. ruderarius* and *B. velox* are sister species supported by PP = 0.99 and bootstrap values = 61 in the BI and ML analyses, respectively. In Cameron et al. (2007), *B. cryptarum* and *B. patagiatus* were sister species (PP = 0.52), and then they attached to *B. moderatus* Cresson (PP = 0.90). However, in our study, *B. crypatarum* and *B. moderatus* are sister species (PP = 1.00; bootstrap values = 97) and their relationship with *B. patagiatus* is distant (Fig. 2). Although most species are strongly supported by bootstrap values in the BI and ML phylogenetic trees based on the six combined genes, there are certain species which are not well supported. For example, *B. longipennis* and *B. affinis* are sister species in the tree but the support values are low (PP = 0.49; bootstrap values = 33). *Bombus longipennis*, *B. affinis*, and *B. franklini* are sister clades, and their support values are also low (PP = 0.27; bootstrap values = 38) (Fig. 2). As shown in Fig. 3, the phylogenetic trees obtained by the MP method showed that there are two distinct clades (short-faced and long-faced) including nearly all subgenera of *Bombus*. This, in general, is consistent with the results obtained using the BI and ML methods, while there were still some variations among subgenera in the topology structure of the phylogenetic trees, and most of the support values on the branches were very low. The phylogenetic tree obtained by the NJ method is different from those resulting from the other three methods (Fig. 5). There are not two distinct clades, and the phylogenetic relationships of some species are not consistent with morphology (Figs 1–3). For instance, *B. kashmirensis*, *B. balteatus* Dahlbom, *B. hyperboreus* Schönherr, *B. neoboreus* Sladen, *B. alpinus* (Linnaeus) and *B. polaris* Curtis belong to the same subgenus, *Alpinobombus*. However, *B. kashmirensis* formed a single clade in the NJ phylogenetic tree. Likewise, *B. haematurus* Kriechbaumer was also removed from the subgenus Pyrobombus and formed a single branch in the NJ phylogeny (Fig. 5). The support values were also low for many bumble bee species in the NJ phylogenetic tree. These results suggest that the BI and ML methods were better than MP and NJ to analyze the phylogenetic relationships among a large number of samples.

**Figure 6.**
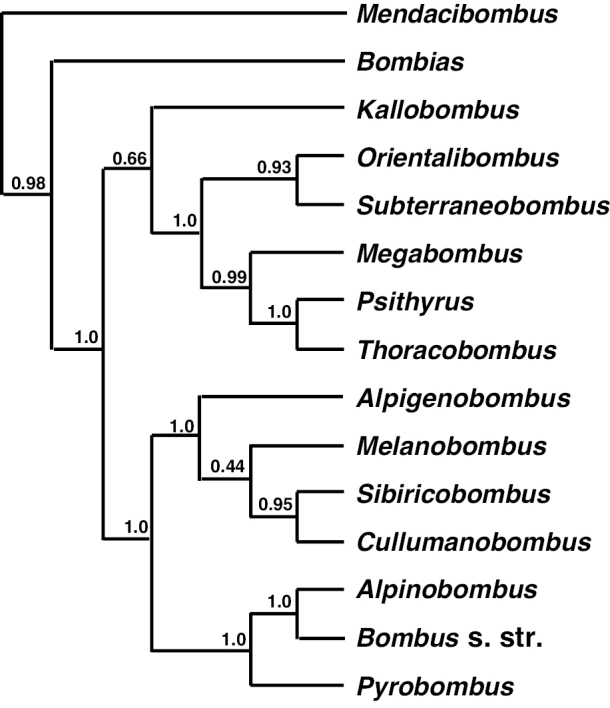
Phylogenetic relationships of the subgenera of *Bombus* from the Bayesian Inference tree (Fig. 2); nearly all of them are well supported by posterior probabilities.

Furthermore, based on the monophyletic groups of bumble bees in the phylogenetic trees of Cameron et al. (2007), morphology, and the important behavioral and ecological characters of bumble bees, Williams et al. (2008) simplified the subgeneric classification of bumble bees from 38 to 15 subgenera. The results of our BI, ML, and MP analyses are consistent with what Williams proposed (Figs 2, 6). Moreover, we constructed the phylogenetic tree by BI based on the combined six genes (Fig. 6), and most clades were well supported by posterior probabilities except the one formed by *Melanobombus* and its sister clade *Sivircobombus* + *Cullumanobombus* (PP = 0.44). It may be that these 15 subgenera are in close proximity in a molecular evolutionary sense, and we need to distinguish them using other information. These results suggest that molecular methods can determine the taxonomic status of the majority of species in *Bombus*, and that it is consistent with morphological identification, but there are a few species in the phylogenetic tree for which the posterior probability and bootstrap values are a little low, and their classification may need to be further supported by combining other criteria with morphology.

### Distinguishing bumble bee species

There are many *Bombus* species distributed in diverse regions all over the world. Previous studies revealed that color pattern and the characters of the male genitalia could clearly distinguish the subgenera of *Bombus* (Franklin 1954; Hines et al. 2006). There have been some problems in some cryptic species complexes; for instance, according to the color pattern, it is easy to consider *B. cryptarum*, *B. lucorum*, and *B. magnus* Vogt as one species, but the chemical and molecular evidence suggests that they are three distinct species (Carolan et al. 2012). Molecular methods are a powerful tool for inference of phylogenetic relationships (Ratnasingham and Hebert 2013). Because the evolutionary rates of single genes are different, each genetic marker has its advantages and disadvantages in phylogenetic analysis and a single gene cannot always clearly resolve the classification of species. Hines et al. (2006) found that combined genes can obtain stronger support values in some nodes compared to individual genes. In the present study, we also explored the power of combining multiple genetic markers which are conserved in evolution and accurately infer phylogenetic relationships of species (Cameron and Mardulyn 2003; Hebert et al. 2003; Magnacca and Brown 2012; Isaka and Sato 2014; Kjer et al. 2014; Schmidt et al. 2015). When multiple genes were combined, we could generate a clearer phylogeny to accurately determine the taxonomy of species by relying on the ability of the individual genes to reconstruct a known phylogeny and a set of genes to accurately infer the phylogenetic relationships of species. Although it is possible to resolve the taxonomy of species within *Pyrobombus* with combined multiple genes, it is still difficult to distinguish among all *Bombus* species.

China has the largest diversity of *Bombus* species in the world (Williams et al. 2010). However, it has been an increasing challenge to effectively protect and utilize the abundant resource of bumble bees in China. Our results significantly enhance our understanding of the taxonomic status and distribution of *Bombus* in China and provide an important foundation in further revealing the evolutionary history of *Bombus* and strengthening the protection of bumble bees as a resource. Some species readily reproduce, so they have been successfully commercially reared for pollination in greenhouses. For example, *B. terrestris* (Linnaeus) (subgenus Bombus s. str.) has been reared commercially in Europe and *B. impatiens* Cresson (subgenus Pyrobombus) has been reared commercially in North America. Although we could introduce these *Bombus* species to our country for pollination of crops in large greenhouses, there is the potential that they could bring new pathogens that would threaten the native species. As a result, the most practical solution would be to identify native species that can be readily reared for large-scale production. Our results showed that *B. longipennis*, *B. minshanensis*, *B. lantschouensis*, and *B. terrestris* belong to the subgenus Bombus s. str. and have a close phylogenetic relationship within the subgenus. These species are widely distributed throughout China, so they may represent an ideal option for commercial rearing in China. Our future goal is to distinguish all species of *Bombus* completely and accurately using a combination of different methods, thereby leading to a better understanding of the distribution and evolutionary history of bumble bees in China and improving our strategies of biodiversity conservation to promote pollinator populations.

## Supplementary materials

Supplementary material 1 Key to the 26 species of the genus *Bombus* Data type key Explanation note Key to the 26 species of the genus *Bombus* for males and females. https://binary.pensoft.net/file/492199This dataset is made available under the Open Database License (http://opendatacommons.org/licenses/odbl/1.0/). The Open Database License (ODbL) is a license agreement intended to allow users to freely share, modify, and use this Dataset while maintaining this same freedom for others, provided that the original source and author(s) are credited.Liu-Hao Wang, Shan Liu, Yu-Jie Tang, Yan-Ping Chen, Jie Wu1, Ji-Lian Li
