# Supplementary material for: Using the combined gene approach and multiple analytical methods to improve the phylogeny and classification of Bombus (Hymenoptera, Apidae) in China
Source: Zookeys. 2020 Dec 30;1007:1–21. doi: 10.3897/zookeys.1007.34105 (PMC7788072; doi:10.3897/zookeys.1007.34105)
Supplement: Supplementary material 1 — Key to the 26 species of the genus Bombus [file zookeys-1007-001-s001.docx]

**Key to the 26 species of the genus *Bombus* for females**

1. Outer surface of the hind tibia is broad and nearly flat with fringed stout hairs, which form a pollen basket (corbicula), the inner distal margin with a comb of stout spines (restellum); Sternum 6 has no ventro-lateral keels……...….………..**2**

- Outer surface of the hind tibia is convex and without a pollen basket; the inner distal margin has a comb of stout spines (rastellum), and with ventro-lateral keels in sternum 6; the black hair of thoracic dorsum can form at least one black band and extend between the wing bases; thorax is mainly black hair, only one band of pale hairs in anterior part; thoracic dorsum is mostly black hairs, only a few pale hairs intermixed in anterior and posterior part…………...……….***Bombus turneri***

1. Outer surface of hind tibia is smooth and shiny…………………………………..**3**

- Outer surface of hind tibia is rough, matt and dull, which has spaced widely with quite long stout hairs near center of the proximal half, the breadth exceed the last tarsal segment. Thoracic dorsum with many black yet grey-tipped hairs, but which has no pale band in anterior part ………………………....***Bombus waltoni***

1. No long hairs near center of the proximal half of hind tibia…………….………..**4**

- At most one or two long stout hairs in the middle of the proximal half of hind tibia……………………………………………………………………………..…**5**

1. Mandible terminal is rounded broadly with two or three teeth. Thoracic and metasomal dorsum hair are yellow or olive-grey with at least one black band……………………………………………………………………………….**6**

- Mandible terminal is not rounded broadly with six evenly-spaced large triangular teeth. In anterior and posterior part of thoracic dorsum has pale bands with grey-white hair. Posterior part of tergum 2 with yellow or white hair. Malar area is much shorter than broad…………………..………………. ***Bombus kashmirensis***

1. Midleg basitarsus without narrow tooth or spine, the distal posterior corner with broadly or narrowly rounded, which form an angle of more than 45°……………**7**

- Midleg basitarsus with a narrow tooth or spine, the distal posterior corner extend to form a acute angle of 45° or less………………………………………….……**8**

1. Thorax hairs are not entirely black…………………………………………….….**9**

- Thorax hairs are entirely black with very few isolated pale hairs scattered in anterior band. Terga 1-5 with some orange-red hair, terga 2-3 with some yellow or red hair, tergum 4 hair is usually extensively black. Legs with long black hair. ‘Tail’ hair is red. The fourth antennal segment is shorter than broad………………………………………………………….***Bombus trifasciatus***

1. Mandible with a notch near its distal posterior part, which is almost as deep as wide separating a strong posterior tooth; the central area of clypeus with strongly swollen and bulging, many dense large punctures; the posterior margin of hindleg basitarsus with broadly and evenly strongly curved……………...………..……**10**

- Mandible with a notch near its distal posterior part, which lack or less than half as deep as wide or not separating a posterior tooth; the central area of clypeus with slightly swollen or nearly flat; large punctures which widely scattered among many smaller punctures; the posterior margin of hindleg basitarsus with evenly weakly curved, or which is strongly in its hindleg basitarsus………………...…**11**

1. Clypeus is flatten, smooth and shining with widely spaced tiny punctures in central area, the larger puntures only at the edges; clypeus without a longitudinal groove marked with punctures in its dorsal median; the cheek length is less than half the eye; Sternum 6 with a shiny and raised median longitudinal keel in its 1/3 posterior …………………………………………………………………………**12**

- Clypeus is bulbous with very few scattered large or medium punctures in central area; the corbicular fringes are longer than the widest of the hindleg tibia, the corbicular surface in hindleg tibia with moderately convex and swollen in anterior part and short of posterior concavity in the distal half; Sternum 2 is slight bulging with a weakly rounded transverse ridge between the anterior and posterior margins; clypeus with convex in dorsal part but without a deep median longitudianal groove………………………...……………………………...……**13**

1. Thorax with a large round white patch in black part between the wing bases. Dorsum of head, tergum 2 and sides of thorax with short black hair. The body length is more than 22mm……………………...…………...……***Bombus festivus***

- Thorax without large round white patch between the wing bases. The body length is less or more than 22mm. Tergum 2 or 3 with a black or red band. Metasomal tail hairs are red, orange-red or pinkish. Tergum 5 hair is white, or pinkish in middle third segment. Tergum 2 hair with extensively red at least in posterior part. The distance between the eye and the mandible is more than twice the proximal breadth of the mandible………………………..………...…….***Bombus supremus***

1. Terga 4-5 hairs are almost white; cheek is obviously shorter than broad; face and thoracic concavity hairs are black or white or yellow; wing colour is brown…..14

- Terga 4-5 hairs are deep red; face hairs are black; tergum 2 and the anterior part of thoracic dorsum hairs are black, sometimes with a few golden yellow laterally; the corbicular fringes of hind tibia hairs are black…………....…..***Bombus ignitus***

1. Clypeus central area with extensively scattered large and small punctures, which is irregular and dull; in the distal half hindleg basitarsus with short slightly feathered hairs widely spaced, and the shining surface is clearly visible……….**15**

- Clypeus central area with scattered mainly small punctures, which is smooth and shiney; in the distal half hindleg basitarsus with dense overlapping short slightly feathered hairs largely covering the shining surface……………………….……**16**

1. Clypeus with sparse micro-punctures, the distance between them are more than twice their breadths; Terga1-2 hairs are widely balck…………………………..**17**

- Clypeus with many micro-punctures, the distance between them are twice their breadths; Terga1-2 hairs are entirely yellow or cream-yellow; Tegra3-4 are black with long white hairs in wide posterior fringes………………***Bombus personatus***

1. Thoracic dorsum hairs are grey or yellow or brown; between wing bases with a spot or distinct black band…………………………………………………….…**18**

- Thoracic dorsum hairs are orange-brown, the anterior part of its with some black hairs; between wing bases with minority or without obviously distinct black ban or spot; the corbicular finges with mainly black hair, sometimes with pale tips in a few hairs; hair of face mostly black; hair of terga3-6 are completely black………………………………………………………...…***Bombus opulentus***

1. Hairs of thoracic dorsum anterior part with pale band are bright lemon yellow or white……………………………………….…………………………………….**19**

- Hairs of thoracic dorsum anterior part with pale band are yellow or cream, the posterior pale ban is broad and the colour is the same as anterior part; lower thoracic concavity hairs intermixed with pale and black……………………………………………………...***Bombus lantschouensis***

1. Hair of thoracic dorsum is entirely black; the colour of legs are mainly orange………………………………………………………....***Bombus flavescens***

- Hair of thoracic dorsum is white that always form a distinct band with black hair between the wing bases; hair of head is mostly grey and the corbicular fringes are orange……………………………………………………………. ***Bombus lepidus***

1. Thoracic dorsum hairs with wide white or yellow bands…………….…………**20**

- Thoracic dorsum hairs are mainly black; Terga3-5 hairs are red...............................................................................................***Bombus pyrosoma***

1. Clypeus central half almost lack the even micro-punctures; thoracic dorsum hair with a black band between the wing bases, the breadth is less or more than the yellow or white band of thorax anterior part; thorax hairs are cream yellow or white; Tergum2 with long hairs are straight and erect over the anterior two thirds, which is curved and more decumbent along the posterior edge; Tergum5 with the longest hairs that exceed the posterior edge, and the length is more than the maximal breadth of hind basitarsus……………..…………..***Bombus difficillimus***

- Clypeus central half with a few sparse punctures; thoracic dorsum hairs are entirely yellow, the breadth of band is less than thorax anterior yellow band; thorax hairs are lemon yellow or straw yellow; Tergum2 with short hairs are straight and erect over the anterior one quarter or less, which has curved and more decumbent along the posterior edge; Tergum5 with the longest hairs that exceed the posterior edge, and the length is less than the maximal breadth of hind basitarsus…………………………………………………….. ***Bombus melanurus***

1. Hairs of tail are bright orange………………………………………..………….**21**

- Hairs of tail are yellow or grey or black; thoracic dorsum hairs are yellow or white with a black ban or spot between the wing bases, few or without black hairs intermixed in the anterior pale band; Terga4-5 hairs mainly yellow or white; Terga2-3 hairs are yellow; face and femora of legs with many black hairs; corbicular fringes and sterna2-5 hairs are black…………..…. ***Bombus filchnerae***

1. Thoracic dorsum hairs with the pale anterior band are bright lemon yellow, and the posterior band with few or no pale hairs, and thoracic lower side with many black hairs intermixed…………………………………………………...………**22**

- Thoracic dorsum hairs with the pale anterior band are white, the posterior pale band broad with the same colour as anterior……………………………...……..**23**

1. Hairs of the face are black, Tergum3 are red……………………………………**24**

- Hairs of the face are often white, Tergum3 mainly black with white ate the sides, thoracic dorsum pale bands with white, Tergum5 with pale orange or pale pink…………………………………………………………..….. ***Bombus sichelii***

1. Hairs of Tergum2 are grey or yellow in anterior part and orange in posterior part, sometimes widely black between anterior and posterior part; Tergum3 hairs with mainly orange; thoracic dorsum with a black band between the wing bases, and the black can extend the median point of posterior part…..... ***Bombus impetuosus***

- Hairs of Tergum2 are yellow with black intermixed laterally, Tergum3 are mainly black; thoracic dorsum with few black band between wing bases………………………………………………..……………***Bombus remotus***

1. Vertex of the head, lateral thorax, thoracic dorsum posteriorly and side of tegum1 with rich long feathery hair intermixed………………………***Bombus longipenni***

- Vertex of the head, lateral thorax, thoracic dorsum posteriorly and side of tegum1 without rich long feathery hair intermixed; in the medial posterior area of tergum2 with rare punctures, and without obvious for intervening surface sculpture…………………………………………………………***Bombus lucorum***

1. Thorax lower side with white hair or intermixed many black hairs; thoracic dorsum with clearly defined black ban between the wing bases, and few white hair intermixed at side; the pale band posteriorly or at the front of the anterior pale band with few black hair intermixed; thorax lower side with half white hair or intermixed with many black hairs…………………..……..***Bombus patagiatus***

- Thorax lower side with white hair or intermixed many black hairs; thoracic dorsum with weakly defined black ban between the wing bases, and dense white hairs intermixed at side……………………………..……..***Bombus minshanensis***

1. Thoracic bands are grey-white…………………………………………………..25

- Thoracic bands are yellow or orange; dorsum and posterior dorsal fringe of head hairs are mainly black and with some short pale hairs intermixed; hairs of mostly tergum2 are yellow, anteriorly with black, tergum3 red; corbicular fringes hairs are predominant black hair with few pale tips; tergum6 posteriorly with notch…………………………………………………………... ***Bombus friseanus***

1. Hairs of thoracic dorsum pale bands are white, the lower half of the side of thorax are black, tergum2 with black or bright yellow anteriorly; the body size and the hair of length are medium………………………….………***Bombus rufofasciatus***

- Hairs of thoracic dorsum pale bands and lower half of the side of thorax are white, tergum2 with white or cream or bright yellow anteriorly; the body size is small and the hair is long……………………………………...….. ***Bombus ladakhensis***

**Key to the 26 species of the genus *Bombus* for males**

1. Gonostylus without the medium length branched hairs at the inner proximal process; gonostylus and volsella highly sclerotised with dark brown; volsella inner margin not always with a process or hooks; penis-valve head is straight or curved………………………………………………………..……………………**2**

- Gonostylus with the medium length branched hairs at the inner proximal process; gonnostylus and volsella weakly sclerotised with pale yellowish; volsella inner margin without a process or hooks; volsella in distal posterior half part is brownish, and which hardly produced the inner corner towards the midline; penis-valve head almost straight; hairs of Tergum5 are orange or red; gonocoxa is narrow to a single pointed from the inner part distally to the end, which narrower near the inner side……………………………………...…………***Bombus turneri***

1. Antenna length is medium to long, which can reach back to the tegula anterior margin of the wing base; penis spatha proximal end with narrowly and sharply pointed; penis-valve head is straight or curved; eyes are enlarged or not enlarged relative to females…………………………………………………………..….…**3**

- Antenna length is medium to long, which not always reach back to the tegula anterior margin of the wing base; penis spatha proximal end with rounded; penis-valve head is straight; eyes are always much bigger than females; thoracic dorsum anterior part without distinct white band, which intermixed black and white hairs; Terga4-6 hairs are black or orange with white tips... ***Bombus waltoni***

1. Penis valve dorso-ventrally is narrow with slightly ventrally curved; anternna length is medium or longer, which can reach or exceed the tegula of the wing base………………………………………………………………………….…….**4**

- Penis valve dorso-ventrally is greatly broadened to form half of a broad tube; anternna length is medium, which can not reach the back of the tegula posterior margin of the wing base…………………………………………………….…….**5**

1. Penis-valve head with a distinct broad hook from the dorsal aspect turned inwards distally…………………………………………………………………………….**6**

- Penis-valve head with almost straight or turned slightly outwards from the dorsal aspect turned inwards distally……………………………...……………………..**7**

1. Hairs of thoracic dorsum pale bands and Terga1-2 are lemon yellow or white, Terga5-6 are white, yellow, pink or brown; penis-valve head with the tip turned outwards from the body midline in dorsal view……………………………..……**8**

- Hairs of thoracic dorsum pale bands and Terga1-2 are golden yellow, Terga5-6 are orange-red; penis-valve head is straight form the dorsal view without turned outwards of the tip from the body midline…………………...….. ***Bombus ignitus***

1. Gonostylus with only a simple triangle and no inner proximal process, in cross section with a submarginal longitudinal groove, and the inner distal margin of gonostylus with a little thickened…………………………………………………**9**

- Gonostylus shape is variable, yet with a obvious inner proximal process; in cross section without a submarginal longitudinal groove, and the inner distal margin of gonostylus is flattened and blade-like…………………………………..……….**10**

1. Ventral part of volsella distal half is broad; the posterior margin of hind tibia outer surface is convex……………………………….………………...………..**11**

- Ventral part of volsella distal half is narrow, the inner hooks are close and point to the distal end; the posterior margin of hind tibia outer surface is concave………………………………………………………………..…………**12**

1. Hairs of thoracic dorsum posterior part black or yellow or white; between eyes and gena posterior and on the scutellum with weakly branched hairs…………..**13**

- Hairs of thoracic dorsum posterior part are black, little branched hairs are grey and the posterior fringe long hairs are yellow; between eyes and gena posterior and on the scutellum with clear thick long branched feathery hairs……………………………………………………….…***Bombus longipennis***

1. Midleg and hindleg tibiae and tarsi are almost black, and fringed hairs are black or yellow; in central part of hindleg tibia outer surface without hair and shiny; thoracic dorsum hairs with a quite obvious black band between the wing bases; hairs of thoracic dorsum anterior part are yellow, Tergum are yellow, Terga4-6 are orange…………………………………………………….….. ***Bombus lepidus***

- Midleg and hindleg tibiae and tarsi areorange-brown, and fringed hairs are orange-yellow; the hindleg tibia outer surface is nearly covered by the midlength orange hairs……………………………………………………***Bombus flavescens***

1. Ventral angle in penis-valve shaft approximately half way along the length, and it is distinct as a pronounced sharp angle or a larger transverse process………….**14**

- Ventral angle in penis-valve shaft approximately half way along the length quite broadly rounded or absent; mid basitarsus outer surface with many medium and long hairs; anternna medium or long and can reach or exceed the tegula at the wing base………………………………………………………………..……….**15**

1. Thoracic dorsum hairs are orange brown, and black hairs are minority or not clearly; between the wing bases without a distinct black band or spot…….……**16**

- Thoracic dorsum hairs are grey yellow or brown; between the wing bases with a distinct black ban or spot……………………………………………...…………**17**

1. Cheek length is more than 1.5× the width of the mandible between the mandibular articulations; volsella posterior inner process much longer than wide; hairs of thoracic dorsum are white with a black band between the wing bases, tail is black or yellow, Tergum3 is bright red; the body size medium……………………………………………………….. ***Bombus supremus***

- Hairs of thoracic dorsum are completely black, in anterior and posterior part with some brown hairs, Tergum3 are black or brown, Tergum5 are orange; antennal segment4 shorter than broad; gonocoxa distally is broad, and form a short broad rectangular strap………………………………………...……***Bombus trifasciatus***

1. Half of anterior in thoracic dorsum anterior pale band without some scattered black hairs……………………………………………………….………………**18**

- Half of anterior in thoracic dorsum anterior pale band with some scattered black hairs……………………………………………………………………...………**19**

1. Head of penis-valve turned inwards distally form an incurved ‘bowl’ or ‘spoon’ shape; ventral angle in penis-valve shaft about half way along the length with a broad transverse paddle-like process…………………………………..………..**20**

- Head of penis-valve turned inwards distally form a dorso-ventrally flattened ‘sickle’ shape; ventral angle in penis-valve shaft about half way along the length with a pronounced angle; thoracic dorsum hairs with a black band between the wing bases, pale bands between wide anterior and posterior; hairs of thoracic dorsum pale bands are white, the length is long……….…..***Bombus kashmirensis***

1. Thoracic dorsum with yellow or white anterior and posterior bands; penis valve with sharply point, and the recurved head is narrow…………………………….**21**

- Thoracic dorsum with evenly brown hairs; the recurved head of penis valve is broad, forming an neatly equilateral triangle………...…………..***Bombus festivus***

1. Hairs of Terga4-5 are entirely black…………………...………***Bombus opulentus***

- Hairs of Terga4-5 are orange or grey, Tergum2 are mainly lemon yellow or with a few black hairs intermixed in posterior part in certain individual, Terga5-6 with orange, thoracic dorsum are lemon yellow intermixed with many black hairs………………………………………………………….…..***Bombus remotus***

1. Terga3-6 hairs are orange……………………………………***Bombus impetuosus***

- Terga3-6 hairs are yellow or grey; thoracic dorsum hairs are grey-white in anterior and posterior bands; legs and Tergum7 hairs are mainly black………………………………………………….………..***Bombus filchnerae***

1. Thoracic dorsum hairs are broad yellow in posterior band, similar to the anterior yellow band in breadth; Tergum5 hairs are yellow or cream; hairs of the body are black without pale tips…………………………...………***Bombus lantschouensis***

- Thoracic dorsum hairs are narrowly yellow in posterior band, the breadth is less than half of the anterior yellow band; Tergum5 hairs are bright white; hairs of the body are black with pale tips……………………………………***Bombus lucorum***

1. Both thoracic dorsum pale bands and Tergum1 hairs are white or cream-white…………………………………………………......……***Bombus patagiatus***

- Both thoracic dorsum pale bands and Tergum1 hairs are yellow………………………………………………..….…***Bombus minshanensis***

1. The sharp dorsal ridge in gonocoxa subside into the convex dorsal surface, so which is rounded and finger like in distal protion…………………….…………**22**

- The sharp dorsal ridge in gonocoxa can reach the distal edge, which is dorsally flat in its inner side distally; hairs of Terga1-2 are yellow, Terga3-6 anterior part are black yet posteriorly are yellow or white; outer proximal corner of penis-valve head with long conical or cylindrical spine……..……..***Bombus personatus***

1. The diatal margin of gonostylus is concave; volsella extending over the gonostylus distally nearly twice with its breadth………………….…………….**23**

- The diatal margin of gonostylus is convex; volsella extending over the gonostylus distally only the same as its breadth……………………………………………..**24**

1. Face hairs are yellow; the narrowest breadth of inner proximal process in gonostylus is one quarter of its length; hairs are short and uniform………………………………………...…………..….***Bombus melanurus***

- Face hairs are black; the narrowest breadth of inner proximal process in gonostylus is as wide as nearly 1/2 length of its; hairs are long and ununiform…………………………………………...………***Bombus difficillimus***

1. Thoracic dorsum with pale bands are grey-white; eyes are obviously bigger than females…………………………………………..…………***Bombus rufofasciatus***

- Thoracic dorsum with pale bands are yellow; eyes are similar with females…25

1. Terga6-7 hairs are orange with white-tipped; the crook head of penis-valve turned back nearly parallel the head and fused to over two thirds of its length………………………………………….……….…….***Bombus ladakhensis***

- Terga6-7 hairs are orange without white-tipped; the crook head of penis-valve free of the head over half of its length; the inner distal process of volsella form a broad short stump……………………………………………...….***Bombus sichelii***

1. Hairs of thoracic dorsum are yellow with a completely black band between the wing bases……………………………………………...………***Bombus friseanus***

- Hairs of thoracic dorsum are yellow with black hairs intermixed between the wing bases, but not forming distinctly black band…………..….***Bombus pyosoma***
